# Emergency department mental health presentations in young children: a retrospective chart review

**DOI:** 10.1111/jpc.16600

**Published:** 2024-06-21

**Authors:** Elyssia M Bourke, Daniela F Say, Anna Carison, Sinead M O'Donnell, Franz E Babl, the Paediatric Research in Emergency Departments International Collaborative (PREDICT) research network

**Affiliations:** ^1^ Emergency Research Group Murdoch Children's Research Institute Melbourne Victoria Australia; ^2^ Department of Critical Care University of Melbourne Melbourne Victoria Australia; ^3^ Grampians Health Ballarat Victoria Australia; ^4^ Royal Melbourne Hospital Melbourne Victoria Australia; ^5^ Emergency Department Royal Children's Hospital Melbourne Victoria Australia; ^6^ Department of Paediatrics University of Melbourne Melbourne Victoria Australia

**Keywords:** emergency medicine, mental health, paediatrics, psychiatry

## Abstract

**Aim:**

To characterise key features of young people presenting to the emergency department (ED) with a mental health complaint when comparing children (aged 7 to 12 years) and teenagers (13 years and greater).

**Methods:**

Retrospective review of all ED mental health presentations in children aged 7–17 years presenting over a 12‐month period in 2018 to a tertiary children's hospital in Victoria, Australia. Univariate analyses were carried out to examine the relationship between children and teenagers and a number of key presentation variables. Odds Ratios (ORs) and 95% Confidence Intervals (CIs) were calculated for ED management outcomes.

**Results:**

There were 1691 ED mental health presentations in 2018. Of these presentations, 407 (24%) were children aged 12 years or less. The remaining 76% (1284) were teenagers. The younger aged cohort were more likely to be male (OR 2.43, CI 1.92–3.08) and have a past history of autism spectrum disorder (OR 1.92, CI 1.45–1.84). They were more likely to have a presenting complaint of acute behavioural disturbance (OR 2.03, CI 1.59–2.60), be physically restrained (OR 2.01, CI 1.18–3.37) and have sedative medication provided (OR 2.87, CI 1.63–5.04). The older aged cohort were more likely to have a past history of depression (OR 0.19, CI 0.12–0.29) and a presenting complaint of intentional self‐poisoning (OR 0.33, CI 0.15–0.65).

**Conclusions:**

Children aged 12 years or less represent one‐quarter of all young people presenting to the ED with a mental health concern. They experience high rates of acute behavioural disturbance and are more likely to require restrictive interventions during their presentation.

## What is already known on this topic

1. The emergency department is a common first contact point for young people with mental health complaints.
2. These presentations are increasing but the focus of research in the past has been on adolescents rather than young children.

## What this paper adds

1. Children aged 12 or younger represent one‐quarter of all mental health presentations.
2. A diagnosis of autism was twice as common in children 12 years or less than in the adolescent population.
3. These younger children were more likely to have restrictive interventions utilised during their ED presentation.

The emergency department (ED) is a common first point of contact for young people with mental health (MH) conditions seeking medical care.^1^ In recent years, the number of people aged less than 18 years of age with MH conditions presenting to the ED has been rising.^2^, ^3^ These young people are more likely to have extended stays in the ED and to represent for care.^4^, ^5^ They also have a higher rate of admission than those with physical health complaints.^6^ A concerted focus on determining the characteristics of this population and their progress through the ED and hospital system is underway across the globe.^3^, ^7^, ^8^, ^9^

To date, the majority of this research has focused on those aged less than 18 years of age as a whole; or specifically on the older adolescent or teenage population. Younger children (aged 12 years or less) are an important cohort whose presentations require a targeted research focus.

This study aimed to describe the key characteristics and management of young people aged 12 years or less presenting to the ED with a MH complaint and determine differences between this younger age cohort and those who were aged 13 years or older.

## Methods

### Design and setting

A retrospective review of the electronic medical records for all ED presentations with MH diagnostic codes, in children aged 7–17 years, presenting over a 12‐month period from 1 January 2018 to 31 December 2018, to the Royal Children's Hospital (RCH) in Melbourne, Australia, was undertaken. This paper represents a planned sub‐group analysis of the overall MH cohort. The RCH is a 385‐bed tertiary paediatric hospital with an annual ED census of 85 347 children (2018) and is a designated MH facility under the Victorian MH Act, servicing a population of over six million people.^10^, ^11^ The MH service consists of the ED, a 16‐bed adolescent (13–18 years) psychiatric inpatient unit and outpatient services.

### Study procedure

**Fig. 1:**
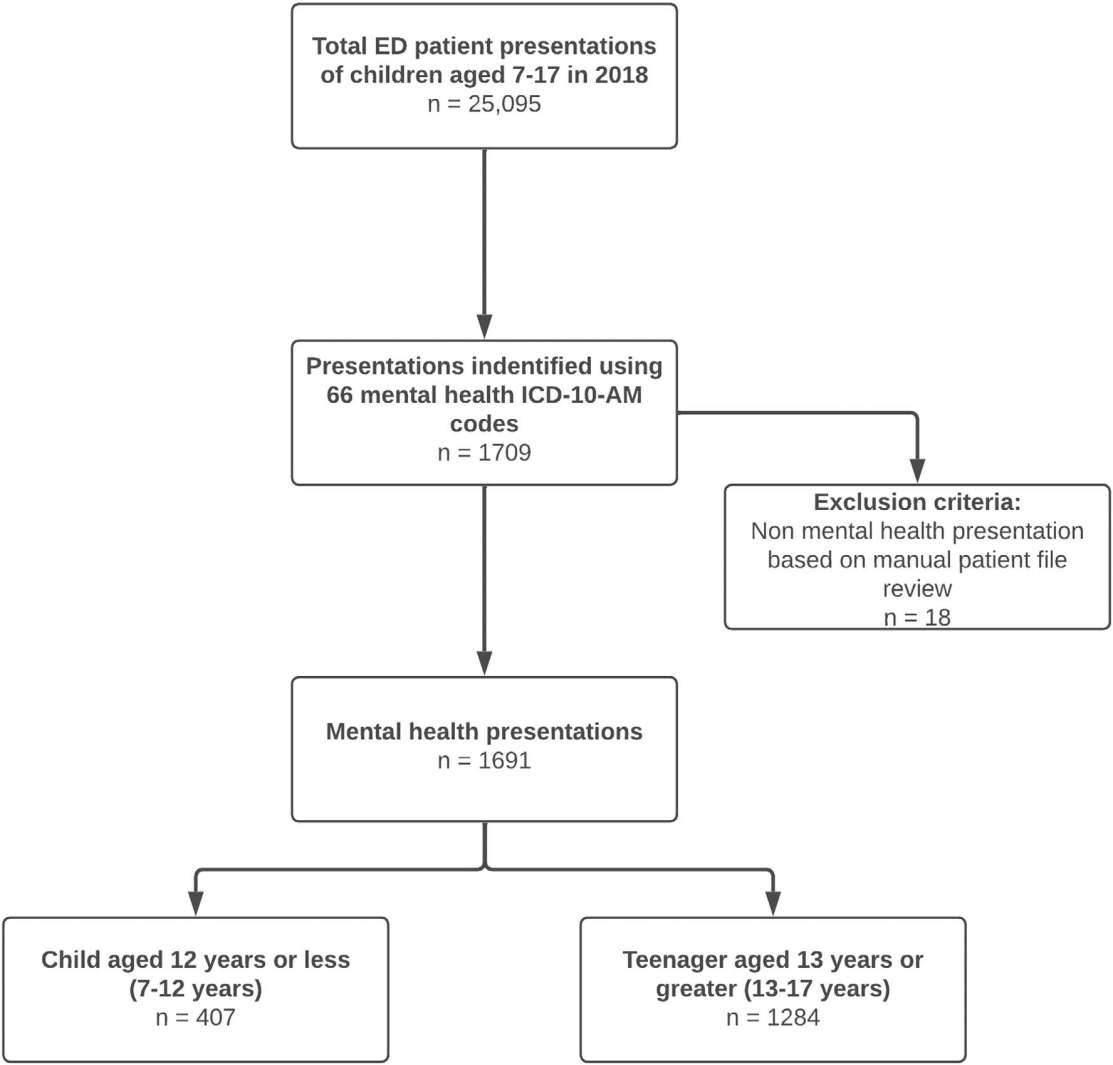
Flowchart identifying children and teenagers presenting to the emergency department with a mental health presentation.

A list of 66 MH‐related International Classification of Diseases, Revision 10, Australian Modification (ICD‐10) diagnostic codes were used to identify ED MH presentations for study inclusion (Table S1). This list was designed to capture all MH presentations and therefore included all MH‐specific diagnostic codes, as well as other diagnoses often associated with MH presentations, for example laceration of wrist and ingestion of foreign body. Each record identified through these ICD‐10 codes was then manually reviewed by DS or AC to confirm that the presentation was MH‐related. All presentations deemed as non‐MH were excluded (Fig. 1).

For each presentation, relevant demographic, diagnostic and management data were extracted. Data extractors were trained and an *a priori* developed coding dictionary was used to ensure consistency. Auditing was conducted throughout the data collection process by three of the study authors: DS, AC and SMOD. Random data checks (or chart reviews) were conducted regularly to ensure data accuracy. Records in which there was uncertainty or discrepancy were also flagged for review and then resolved with senior authors.^12^

### Statistical analysis

Data were entered into an Excel database (Microsoft, Seattle, WA, USA), de‐identified and then exported into Stata 17 for analysis (StatCorp, College Station, TX, USA). Univariate analyses were carried out to examine the relationship between age (7 to 12 years compared with those aged 13 to 17 years) and key presentation variables. Odds Ratios (ORs) and 95% confidence intervals (CIs) were calculated.

### Definitions

- High acuity was defined as an Australasian Triage Scale (ATS) category of 1 or 2. An ATS category of 1 represents an immediately life‐threatening condition requiring medical review on arrival, whilst an ATS category of 2 represents an imminently life‐threatening condition or the need for time‐critical treatment, requiring medical review within 10 min of arrival.^13^
- An ‘acute crisis team response’ is an emergency alert used at RCH (locally this is referred to as a Code Grey) when a patient has escalating behaviour that poses a risk to themselves or others.^10^ It results in a team of medical, nursing, security and allied health staff attending to provide urgent intervention to de‐escalate the patient's behaviour.
- Section 351 provides police with the power under the Victorian Mental Health Act to apprehend a person who appears to have a mental illness to prevent serious or imminent harm to them or others.^11^ Following their apprehension, the person must be taken to a registered medical or MH practitioner to be examined. This commonly involves the individual being brought to the ED for assessment.

## Results

During 2018, there were 25 095 total patient presentations of children aged 7–17 years to the RCH ED, of which 1691 were MH related (7% of total ED presentations in the 7–17 year age group). Within this group, 407 (24%) were children aged 12 years or less. The remaining 1284 (76%) were teenagers aged 13 years or greater (Fig. 1).

### Patient characteristics

Children aged 12 years or less were more likely to be male than their teenage counterparts (OR 2.43, CI 1.92–3.08) and were less likely to have received psychiatric care in the community prior to presentation to the ED (OR 0.37, CI 0.29–0.48) (Table 1). Those 12 years or younger were more likely to present on a weekday than a weekend (OR 1.37, CI 1.02–1.84).

**Table 1:** Demographic and presentation variables of children (7–12 years) and teenagers (13–17 years)

	Mental health presentation in those aged 12 years or less		Mental health presentation in those aged 13 years or greater		Comparison of those aged 12 years and less to those aged 13 years or greater
	*n* = 407		*n* = 1284		
	*n*	%	*n*	%	OR (95% CI)
Sex					
Male	199	49	362	28	M:F 2.43 (1.92–3.08)
Female	208	51	922	72	
Guardianship					
Parent	365	90	1108	86	Parent: non‐parental guardianship 1.27 (0.88–1.87)
Non‐parental guardianship	42	10	162	15	
Unknown	0	0	12	<1	
Past history factors†					
Autism spectrum disorder	107	26	201	16	1.92 (1.45–2.53)
Attention deficit hyperactivity disorder	15	4	76	6	0.61 (0.32–1.08)
Obsessive compulsive disorder	3	1	18	1	0.52 (0.10–1.80)
Borderline personality disorder	1	<1	110	9	0.03 (0.00–0.15)
Eating disorder	4	1	35	3	0.35 (0.09–1.00)
Post‐traumatic stress disorder	9	2	46	4	0.61 (0.26–1.27)
Anxiety	35	9	238	19	0.41 (0.28–0.60)
Depression	23	6	310	24	0.19 (0.12–0.29)
Suicidal ideation	6	1	106	8	0.17 (0.06–0.38)
Self‐harm	12	3	125	10	0.28 (0.14–0.52)
Intellectual disability	2	<1	28	2	0.22 (0.03–0.89)
Schizophrenia/Psychosis	2	<1	28	2	0.22 (0.03–0.89)
Gender dysphoria	2	<1	17	1	0.37 (0.04–1.56)
Recreational substance use	0	0	19	1	N/A
Prior psychiatric community care					
Yes	264	65	1062	83	Yes:No 0.37 (0.29–0.48)
No	140	34	210	16	
Unknown	3	<1	12	1	
Arrival day					
Weekday	332	82	981	76	Weekday:Weekend 1.37 (1.02–1.84)
Weekend	75	18	303	24	
Arrival time					
Arrival in‐hours (0800–1659)	188	46	574	45	In:After hours 1.06 (0.84–134)
Arrival after‐hours (1700–0759)	219	54	710	55	
Means of arrival					
Private car/self/public transport	261	64	777	61	Private Transport:Ambulance/Police 1.17 (0.92–1.48)
Ambulance (with or without police)	81	20	292	23	
Police alone	65	16	215	17	
Brought to hospital under Section 351‡					
No	359	88	1100	86	Yes:No 0.82 (0.57–1.16)
Yes	48	12	180	14	
Unknown	0	0	4	<1	
Attended with					
Parent	144	35	489	38	Parent:Other 0.88 (0.70–1.12)
DHHS§/social worker/psychologist	5	1	20	2	
Alone	258	63	775	60	
Triage category (Cat)					
1 or 2	24	6	100	8	Cat1&2:Cat3–5 0.74 (0.45–1.19)
3 or 4 or 5	383	94	1184	92	
Reason for presentation					
Acute behavioural disturbance	154	38	296	23	2.03 (1.59–2.60)
Anxiety	82	20	198	15	1.38 (1.03–1.86)
Self‐harm	17	4	75	6	0.70 (0.38–1.22)
Suicidal ideation	86	21	437	34	0.52 (0.39–0.68)
Psychosocial crisis	9	2	16	1	1.79 (0.69–4.34)
Eating disorder	13	3	54	4	0.75 (0.37–1.41)
Somatoform disorder	4	1	13	1	0.97 (0.23–3.16)
Intentional self‐poisoning	10	2	90	7	0.33 (0.15–0.65)
Depression	2	<1	29	2	0.21 (0.02–0.85)
Hallucinations	8	2	18	1	1.41 (0.53–3.44)
Acute psychosis	4	1	18	1	0.70 (0.17–2.14)
Other	18	4	40	3	N/A

† Young people could have more than one past history factor, so overall percentages will be greater than 100%.

‡ Victoria's Mental Health Act, Section 351 provides police with the power to apprehend a person who appears to have a mental illness to prevent serious or imminent harm to the person or others. Following their apprehension, they must arrange for the person to be taken to a registered medical practitioner or mental health practitioner to be examined. This commonly involved the individual being brought to the ED for assessment.

§ Department of Health and human Services (DHHS).

The younger aged cohort were more likely to have a past history of autism spectrum disorder (OR 1.92, CI 1.45–2.53) and less likely to have a past history of anxiety (OR 0.41, CI 0.28–0.60), depression (OR 0.19, CI 0.12–0.29), suicidal ideation (OR 0.17, CI 0.06–0.38), self‐harm (OR 0.28, CI 0.14–0.52), an intellectual disability (OR 0.22, CI 0.03–0.89) or psychosis (OR 0.22, CI 0.03–0.89).

Patients aged 12 years or less were more likely to present due to acute behavioural disturbance (OR 2.03, CI 1.59–2.60) or anxiety (OR 1.38, CI 1.03–1.86) than those who were older. They were less likely to present due to suicidal ideation (OR 0.52, CI 0.39–0.68), intentional self‐poisoning (OR 0.33, CI 0.15–0.65) or depression (OR 0.21, CI 0.02–0.85).

### Emergency department progress

During their ED stay, the younger aged cohort were more likely to require an acute crisis team response (OR 1.66, CI 1.12–2.43), be physically or mechanically restrained (OR 2.01, CI 1.18–3.37) and have sedative medication provided (OR 2.87, CI 1.63–5.04) (Table 2). Despite having higher utilisation of these restrictive interventions, those aged 12 years or less stayed in the ED for a shorter time (median 3.4 h compared to 4.0 h), were more likely to be discharged from the hospital (OR 3.79, CI 2.63–5.59) and to have no follow up arranged (OR 2.78, CI 1.38–5.54).

**Table 2:** Emergency department management and disposition of children (7–12 years) and teenagers (13–17 years)

	Mental health presentation in those aged 12 years or less		Mental health presentation in those aged 13 years or greater		Comparison of those aged 12 years and less to those aged 13 years or greater
	*n* = 407		*n* = 1284		
Acute crisis team response in ED	*n*	%	*n*	%	OR (95% CI)
No	359	88	1189	93	Yes:No 1.66 (1.12–2.43)
Yes	47	12	94	7	
Unknown	1	<1	1	<1	
Physical restraint	*n*	%	*n*	%	OR (95% CI)
No	379	93	1240	97	Yes:No 2.01 (1.18–3.37)
Physical	13	3	14	1	
Mechanical	14	3	30	2	
Seclusion	*n*	%	*n*	%	OR (95% CI)
No	391	96	1255	98	Yes:No 1.90 (0.95–3.70)
Yes	16	4	27	2	
Unknown	0	0	2	<1	
Medication provided	*n*	%	*n*	%	OR (95% CI)
No	380	93	1253	98	Yes:No 2.87 (1.63–5.04)
Yes	27	7	31	2	
Medication route (of those receiving medications)	*n* = 27		*n* = 31		OR (95% CI)
Oral	17	63	17	55	Oral:Intramuscular 1.40 (0.43–4.60)
Intramuscular	10	37	14	45	
Intravenous	0	0	0	0	
Length of stay (hours)					OR (95% CI)
Median (minimum–maximum)	3.4 (0.5–22.7)		4.0 (0.08–48.9)		
Disposition	*n*	%	*n*	%	OR (95% CI)
Home	340	84	826	64	Discharged:Admitted 3.79 (2.63–5.59)
Admitted to hospital	37	9	353	27	
Discharged to residential care	24	6	68	5	
Care of relative	3	1	15	1	
Police custody	1	<1	9	1	
Unknown	0	0	1	<1	
Absconded	2	<1	11	1	
Follow up arrangements	*n*	%	*n*	%	OR (95% CI)
No follow up	18	4	21	2	No follow up:follow up 2.78 (1.38–5.54)
Community psychiatry	280	69	1028	80	
General practitioner	33	8	58	5	
Transfer to another unit	12	3	43	3	
Paediatrician	48	12	72	6	
Unknown	11	3	33	3	
Return to ED	5	1	25	2	

## Discussion

To our knowledge, this is the first study to compare the key features of children 12 years of age or less to teenagers aged 13 years or greater who presented to a paediatric ED with a MH problem. Our data provides an overview of the important differences between these age cohorts and will allow for age‐specific management strategies to be designed to improve the care provided to young people presenting to the ED with a MH complaint.

Our data demonstrate that a quarter of paediatric patients presenting to the ED with a MH complaint are aged 12 years or less. This percentage is similar to previous literature, which estimates the proportion of these pre‐teen patients as being between 20% and 30% of all paediatric ED MH presentations.^3^, ^14^ The population prevalence of MH disorders in this younger age group is considerable; with one Australian study revealing 17% of all males and 11% of all females aged 11 years or less in the general population met diagnostic criteria for at least one MH disorder.^15^ These findings reinforce the importance of a concerted research focus on understanding the key factors driving MH presentations to the ED in younger children.

Our study revealed that this younger age cohort were more likely to be male when compared to their teenage counterparts. In fact, those in the 12 years and less age group had a male to female split that mirrored population averages, with 49% of presentations being female and 51% being male.^16^ This is important information, as it adds further evidence to the fact that the female preponderance in paediatric MH presentations which has previously been widely reported does not occur until the teenage years.^3^, ^14^

These younger patients were significantly more likely to present to the ED on a weekday than they were on a weekend, although the majority of these presentations did occur outside of school hours. It has previously been suggested that this could relate to the school environment exacerbating the child's MH condition or alternatively the supervising adult within the school environment being more likely to either recognise or be less able to manage the child's MH condition.^17^

Autism Spectrum Disorder (ASD) had a higher prevalence in the younger age group in our study. Those aged 12 years or less were eight times more likely to have a diagnosis of ASD than the general population.^18^ This is an important diagnostic label within the paediatric MH population, as those young people with ASD presenting to the ED with a MH complaint have previously been demonstrated to experience higher rates of behavioural disturbance, have an acute crisis team response activated more often, be physically restrained and receive sedative medication during their presentation more often than those without this diagnosis.^19^, ^20^

Acute behavioural disturbance was the most common presenting complaint in those aged 12 years and less, accounting for 38% of all MH presentations in this age cohort. Young people with behavioural disturbance pose significant physical and psychological risks to themselves and those caring for them.^21^ A stepwise approach is endorsed when managing young people with behavioural disturbance.^22^

This approach includes using non‐pharmacological strategies such as verbal de‐escalation, a quiet space, offering distraction and involving family or friends when appropriate. In a small proportion of patients, these techniques are not successful, requiring medication or other restrictive interventions such as seclusion or physical and/or mechanical restraint to be utilised to ensure patient and staff safety.^10^, ^23^ In our study, the activation of the acute crisis team as well as the use of physical and mechanical restraints and administration of sedative medication were all more common in the younger age cohort.

Younger patients were more likely to be discharged from hospital than their teenage compatriots and were less likely to be provided with MH follow up. This is an important finding, as a MH disorder has the potential to significantly impact the young person's development and adversely affect their family unit.^15^

The reason for the differences in follow up are unknown at this time. Whilst there may be specific local issues or it may be that older youths are already linked in with existing MH services, it is possible that this reflects youth MH services in general being more geared towards care of the adolescent patient.

This may also be a reflection of the fact that those young people presenting with neurodevelopmental disorders including ASD, which were more common in this younger cohort, are receiving follow‐up through alternative avenues which have not been captured in our dataset.

This study suggests that there are key differences between younger children aged 12 years of age or less and teenagers aged 13 years or greater presenting to the ED with a MH complaint. Further prospective studies are warranted to understand more about these younger children presenting to the ED with MH complaints, to ensure that the MH care provided to them is age‐specific.

This study has limitations. As a retrospective study, a number of the data points collected were reliant on clinician documentation during the clinical encounter. However, we tried to optimise data collection by following recommended guidance.^12^ This study was a single centre study in a metropolitan tertiary paediatric ED, which may limit the generalisability of the results. Also, as our study excluded patients six years of age or younger, it is possible some MH presentations for younger children were missed.

## Conclusions

Children aged 12 years or less represent one‐quarter of all young people presenting to the ED with a MH concern. They are more likely to be male and have a history of ASD than teenagers with MH presentations. They experience high rates of acute behavioural disturbance and are more likely to require restrictive interventions during their presentation than their teenage counterparts. Further research is required to optimally manage this cohort using the least restrictive methods possible.

## Supporting information


**Table S1:** List of 66 International Classification of Diseases, Revision 10, Australian Modification (ICD‐10‐AM) Diagnostic Codes used for patient inclusion.
