# Supplementary material for: Emergency department mental health presentations in young children: a retrospective chart review
Source: J Paediatr Child Health. 2024 Jun 21;60(9):415–21. doi: 10.1111/jpc.16600 (PMC11656674; doi:10.1111/jpc.16600)
Supplement: Supplementary file 1 — Table S1: List of 66 International Classification of Diseases, Revision 10, Australian Modification (ICD‐10‐AM) Diagnostic Codes used for patient inclusion. [file JPC-60-415-s001.docx]

**Supplementary Information**

**Supplemental Table 1: List of 66 International Classification of Diseases, Revision 10, Australian Modification (ICD-10-AM) Diagnostic Codes used for patient inclusion**

| **ICD-10-AM Mental Health Diagnostic Codes for patient inclusion** |
| --- |
| Accidental drug overdose  Accidental paracetamol overdose  Acute anxiety  Acute depression  Acute drug overdose  Acute post-traumatic stress disorder  Acute stress disorder  Affective bipolar disorder  Anorexia  Anorexia nervosa  Anxiety disorder  Behaviour disorder  Behavioural disorder  Borderline personality disorder  Chronic depression  Depression with suicidal ideation  Destructive behaviour  Drug addiction  Drug dependence  Drug ingestion  Drug overdose, multiple drugs  Drug withdrawal  Drug-induced mental disorder  Emotional crisis  Emotional disorder  Emotionally unstable borderline personality disorder  Hallucination  History of self-harm  Hysteria  Intentional drug overdose  Intentional paracetamol overdose  Manic depression  Manic disorder  Mental and behavioural disorders due to use of alcohol, acute intoxication  Mental disorder  Mental health disorder  Multiple drug overdose  Non-accidental drug overdose  Overdose of medication  Overdose of psychotropic  Overdose of sedative or hypnotic  Overdose of tricyclic  Paracetamol overdose  Paracetamol overdose of undetermined intent  Persistent mood disorder  Post-traumatic stress disorder  Psychiatric disorder  Psychosomatic disease  Psychosomatic disorder  Reactive psychosis  Schizophrenia  Social problem  Somatoform disorder  Stress and adjustment reaction  Suicidal ideation  Suicidal risk  Suicidal thoughts  Suicide by hanging  Suicide ideation |
| **Additional ICD-10-AM Diagnostic Codes for patient inclusion** |
| Laceration of upper arm  Laceration of wrist  Forearm laceration  Wrist laceration  Ingestion of foreign body  Swallowed foreign body |
